# Silicon–Organic Hybrid Electro-Optic Modulator and Microwave Photonics Signal Processing Applications

**DOI:** 10.3390/mi14111977

**Published:** 2023-10-25

**Authors:** Zihan Zhou, Meng Chao, Xinxin Su, Shuanglin Fu, Ruonan Liu, Zhihua Li, Shuhui Bo, Zhuo Chen, Zhenlin Wu, Xiuyou Han

**Affiliations:** 1 School of Optoelectronic Engineering and Instrumentation Science, Dalian University of Technology, Dalian 116024, China; zhzhou@mail.dlut.edu.cn (Z.Z.); chaomeng@mail.dlut.edu.cn (M.C.); xinxinsu@mail.dlut.edu.cn (X.S.); fushuanglin@mail.dlut.edu.cn (S.F.); zhenlinwu@dlut.edu.cn (Z.W.); 2 Institute of Microelectronics, Chinese Academy of Sciences, Beijing 100029, China; liuruonan@ime.ac.cn (R.L.); lizhihua@ime.ac.cn (Z.L.); 3 Engineering Research Centre of Photonic Design Software, Ministry of Education, Beijing 100081, China; boshuhui@muc.edu.cn; 4 Binzhou Institute of Technology, Binzhou 256600, China

**Keywords:** electro-optic modulator, silicon photonics, silicon–organic hybrid, Mach–Zehnder modulator, slot waveguide

## Abstract

Electro-optic modulator (EOM) is one of the key devices of high-speed optical fiber communication systems and ultra-wideband microwave photonic systems. Silicon–organic hybrid (SOH) integration platform combines the advantages of silicon photonics and organic materials, providing a high electro-optic effect and compact structure for photonic integrated devices. In this paper, we present an SOH-integrated EOM with comprehensive investigation of EOM structure design, silicon waveguide fabrication with Slot structure, on-chip poling of organic electro-optic material, and characterization of EO modulation response. The SOH-integrated EOM is measured with 3 dB bandwidth of over 50 GHz and half-wave voltage length product of 0.26 V·cm. Furthermore, we demonstrate a microwave photonics phase shifter by using the fabricated SOH-integrated dual parallel Mach–Zehnder modulator. The phase shift range of 410° is completed from 8 GHz to 26 GHz with a power consumption of less than 38 mW.

## 1. Introduction

Integration is an inevitable trend in future photonics technology development, which can greatly reduce the size, weight, and power consumption (SWaP) of the system. As a key component in highly integrated optoelectronic systems, electro-optic modulators (EOMs) have great potential for applications in fields such as optical communications [1,2] and microwave photonics [3,4,5,6,7,8,9]. The high-performance EOMs should simultaneously have large electro-optic (EO) response bandwidth, small half-wave voltage *V*_π_, and short device length *L*. The common EOMs can be divided into three categories according to the materials: LiNbO_3_ (LN), Si, and EO polymer. LN modulators are the most general and mature EO modulators in current fiber-optical communication systems [10]. And the recent developed thin-film LN modulators can be integrated with silicon photonic platform, showing wide bandwidth and low half-wave voltage [11,12,13,14]. Integrated EOMs based on silicon achieving EO modulation function by free-carrier dispersion effect have good potential because of strong optical field confinement of silicon waveguide and complementary metal–oxide–semiconductor (CMOS) compatibility [15,16,17,18]. With the rapid development of silicon photonics, it has become a major commercial photonics platform for integrated systems. In addition to Si and LiNbO_3_, another potential material for EOMs is EO polymer. Through the optimal design of molecule structure in organic EO material, the ultra-high EO coefficient can be achieved, which has attracted increasing attention in past years [19,20,21]. 

Silicon–organic hybrid (SOH) platform combines the advantages of high integration of silicon photonics and strong linear electro-optic effect of organic material. SOH-integrated modulators can achieve large bandwidth, low half-wave voltage, and short device length at the same time. With the rapid development of fabrication craft, the performance of SOH-integrated EOMs is being enhanced greatly. In 2009, J. Leuthold’s group verified the feasibility of SOH modulators for the first time [22]. Christian Koos’s group has conducted a lot of research on SOH modulator design, fabrication [23,24], and applications [25,26]. They carried out a detailed theoretical study of SOH modulator based on Slot waveguides and greatly expanded the bandwidth of the modulator in experiments. In addition, Shiyoshi Yokoyama’s group investigated the thermal stability of SOH modulators for harsh environments [27,28]. The performance of SOH modulators is becoming increasingly superior through the in-depth exploration of SOH modulators by researchers.

In this paper, we carry out a comprehensive and in-depth study on SOH EOM. The EOM structure design, Slot silicon waveguide fabrication, on-chip organic electro-optic material poling, and the characterization of EO modulation response are investigated comprehensively. The performance of the fabricated MZM is measured and characterized with 3 dB bandwidth of over 50 GHz and half-wave voltage length product of 0.26 V·cm. Furthermore, we have developed an SOH-integrated dual parallel Mach–Zehnder modulator for application in microwave photonics signal processing systems. We have optimized the phase shifting scheme for microwave photonics and performed functional testing and analysis of the phase shifter. The phase shift voltage is controlled in the 0–14 V range, achieving a high-frequency flatness phase shift from X-band to K-band (8–26 GHz) of more than 410° with a power consumption of less than 38 mW. The measured results demonstrate the feasibility of the developed electro-optic modulator for application in microwave photonics signal processing.

## 2. Modulator Design and Device Fabrication

**Figure 1:**
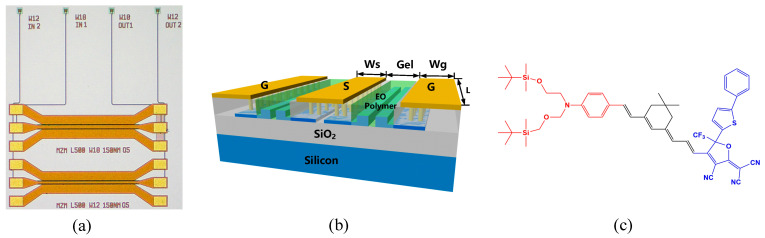
(**a**) Micrograph of the fabricated MZM; (**b**) 3D diagram of a silicon–organic hybrid MZM; (**c**) molecular structure of EO organic material.

The schematic structure of the SOH Mach–Zehnder modulator (MZM) is shown in Figure 1. The MZM comprises two SOH phase shifters driven by coplanar waveguide electrodes. Silicon Slot waveguide is formed by two silicon rails with a narrow slot filled by EO organic material, as shown in Figure 1b. The EO polymer is a guest–host doped system and prepared by the Institute of Physics and Chemistry of the Chinese Academy of Sciences [29]. The chromophores are dissolved, followed by doping into the polymer matrix, functioning as guest. Host material is polymethyl methacrylate (PMMA). The molecular structure of CLD chromophore is shown in Figure 1c. The electron-giving ability of the donor in the chromophore and the electron-absorbing ability of the acceptor are very strong, so it has a good molecular first-order hyperpolarizability (microscopic electro-optical activity). The acceptor part of the CLD chromophore molecule is to introduce additional groups in the furan ring of tricyanofuran (TCF), which increases the electron absorbing ability of TCF receptor, which is beneficial to improve its EO properties, but also increases the difficulty of synthesis. At the same time, the middle conjugated π–electron bridge uses isophorone with high electron transport capacity, which makes the performance of the whole structure very excellent. The absorption loss of EO polymer at 1550 nm is about 3.6 dB/cm. The refractive index of the polymer film was measured as 1.72 using Metricon Prism Coupler Model 2010/M (Metricon, Piscataway, NJ, USA).

The dielectric constants of Si (12.0) and organic polymer (2.9) differ significantly at 1550 nm. And the dielectric constant discontinuity on the sidewall of Slot waveguide makes the optical field confined within the Slot strongly. For the modulation electric field, the electric field can be well applied on the two sides of the Slot waveguide due to the doping of the silicon slab as well. In other words, the optical field and the modulation electric field can be confined within the same Slot region to obtain high modulation efficiency.

**Figure 2:**
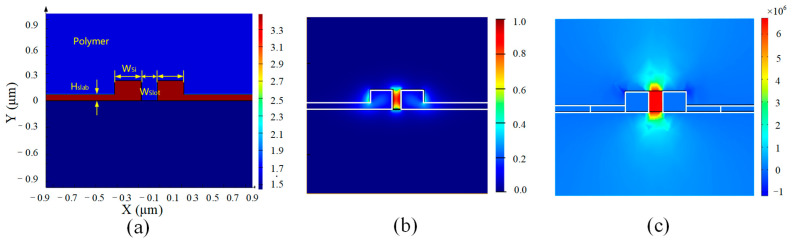
(**a**) Schematic diagram of the structure of Slot waveguide; (**b**) Slot waveguide optical field; (**c**) Slot waveguide modulated microwave electric field distribution.

**Figure 3:**
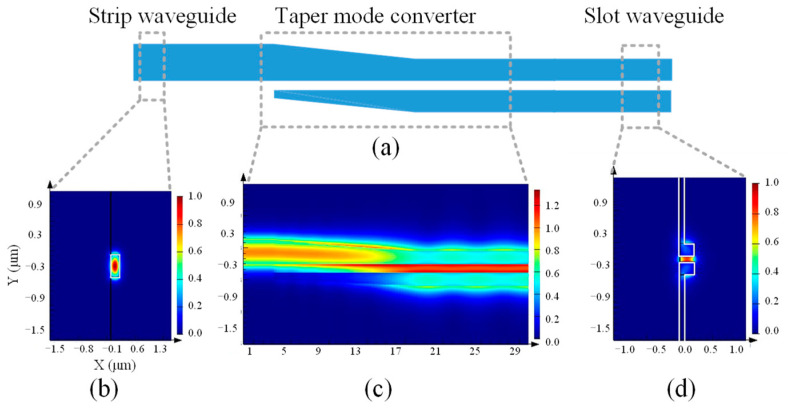
(**a**) Schematic of Strip-to-Slot mode converter; (**b**) optical field distribution in Strip waveguide (Gauss-like mode); (**c**) transmission field distribution of Taper mode conversion; (**d**) optical field distribution in Slot waveguide (non-Gauss-like mode).

The structure of the Slot waveguide and the distribution of the optical and electric field modes in the Slot structure are given in Figure 2. As can be seen in Figure 2b, the optical field distribution of the Slot waveguide is different from that of the general strip waveguide. The field distribution in Strip waveguides is Gauss mode and in Slot waveguides is non-Gauss mode, which will induce large coupling loss when the two waveguides are connected directly. Therefore, the Strip-to-Slot mode converter is designed to reduce the mode conversion loss from Gauss-like mode to non-Gauss-like mode. The structure and the transmission optical field of the Strip-to-Slot mode conversion are shown in Figure 3. By optimizing the design of the Strip-to-Slot mode converter, the coupling efficiency can reach 99.31%.

**Figure 4:**
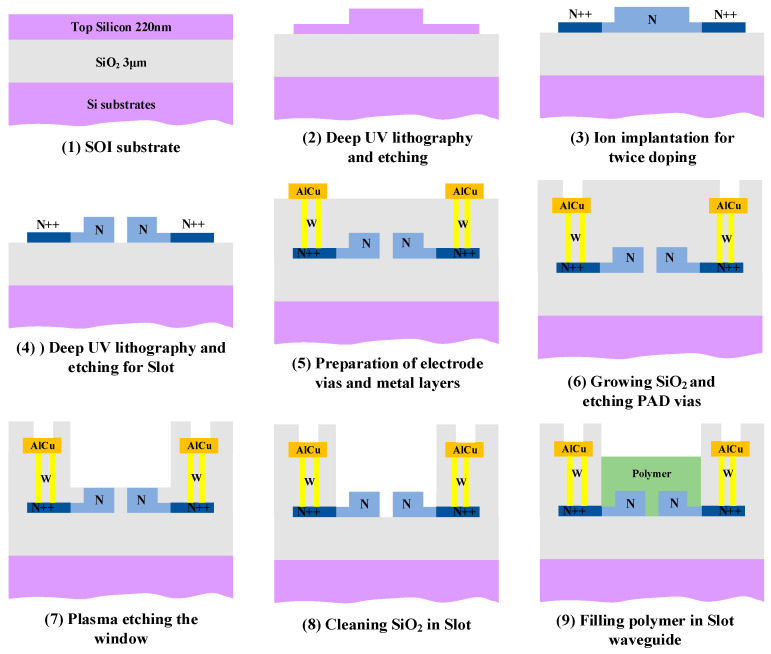
Process flow of SOH modulator fabrication.

Figure 4 shows the main nine steps of the fabrication process of SOH-integrated EOM. The modulators were fabricated by the Institute of Microelectronics of the Chinese Academy of Sciences (Beijing, China). Silicon-on-insulator wafers were used, with 220 nm silicon on top of 3 μm silicon dioxide. The silicon waveguides were processed using deep ultraviolet lithography followed by etching. Through Si etching with 220 nm, 70 nm, and 150 nm height, respectively, the gratings, multimode interferometers, and strip waveguides were obtained. Through deep ultraviolet lithography and Si etching with a height of 220 nm, the fabrication of the Slot waveguide was completed. The width of Slot is 150 nm and the width of the Si rails beside Slot is 250 nm. In order to direct the electric field from the upper metal electrode to both sides of the Slot waveguide, silicon needs to be doped. Low-concentration carrier doping is used near the Slot waveguide, where the optical mode field distribution is concentrated to reduce the optical absorption loss, and high-concentration carrier doping is used in the slab area, where the optical mode field distribution is sparse to increase the conductivity of the slab and reduce the resistance. According to the frequency response determined by the *RC* constant, an appropriate increase in doping concentration will increase the bandwidth of the modulator to a certain extent. Therefore, we used graded doping to improve conductivity while avoiding significant optical loss due to doping. The silicon near Slot is n-doped with a concentration of 3 × 10^16^ cm^−3^, while the concentration further away is 1 × 10^20^ cm^−3^. In step (7) and (8), we first used a dry etch of 1.2 μm and then a wet etch for 3 min to remove the silicon oxide from the Slot waveguide without damaging the rest of the structure.

**Figure 5:**
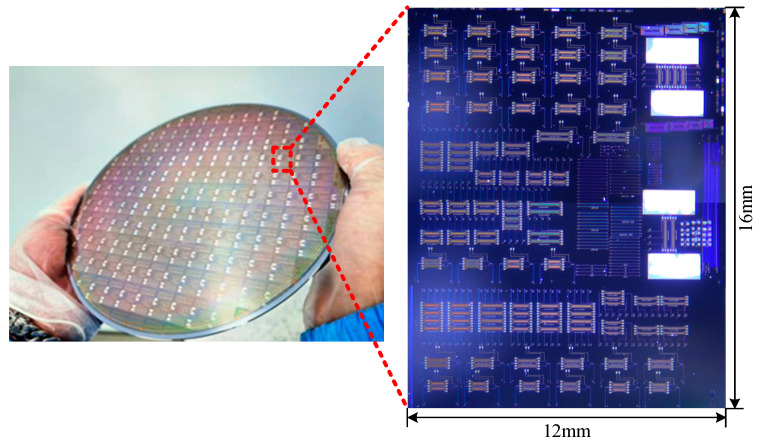
The modulator chips fabricated on an eight-inch wafer and micrograph of single block with size of 12 mm × 16 mm.

After completing the preparation of the waveguide and electrode based on steps (1)–(8) in Figure 4, the 8-inch wafer made with modulator structures is shown in Figure 5, with a single block size of 12 mm × 16 mm.

**Figure 6:**
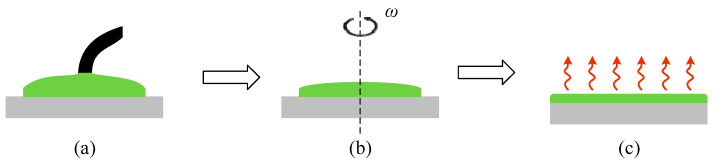
Schematic of spin-coating: (**a**) dropwise addition of polymer solution; (**b**) spin-coating of polymer solution to form a film; (**c**) heating and drying to remove solvent.

A rotational coating method is used to fill the EO organic polymer into the Slot uniformly and sufficiently, as shown in Figure 6. First, the CLD chromophore is doped to PMMA at a concentration of 50 wt%, and both are dissolved into organic solvent. The chip to be spin-coated is placed in the spin-coater (Laurell, WS-400Bz-6NPP-Lite, Lansdale, PA, USA), and then a certain amount of polymer solution is added dropwise to the device surface. The chip with EO polymer on the surface is first spin-coated at 500 rpm for 10 s and then rotated at 1500 rpm for 30 s in the spin-coater to form a thin and uniform film on the surface of the device. After spin-coating, the chip is placed on a heating plate at 70 °C (Bakon, BK946S, Shenzhen, Guangdong, China) for 15 min to remove most of the solvent and release the stress from the polymer chain segments. The chip is then placed in a vacuum drying oven for 12 h with a vacuum of less than −0.09 MPa and a temperature of 60 °C to ensure that the organic solvent is completely evaporated and will not affect the subsequent operations.

**Figure 7:**
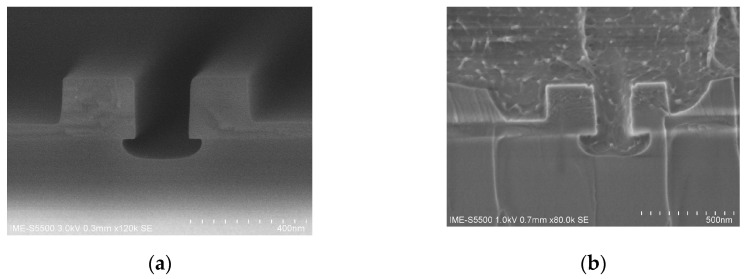
SEM of Slot waveguide cross-section: (**a**) without spin-coating EO polymer; (**b**) with spin-coating EO polymer. The two SEM photos were obtained with different chip samples.

Figure 7 shows the SEM images of the Slot waveguide cross-section before and after spin-coating polymer. As can be seen from Figure 7b, the overall EO polymer filling in the Slot waveguide is complete, uniform, sufficient, and with no bubbles. It can also be seen that, in the process of Slot structure etching, the SiO_2_ under the Slot waveguide is hollowed out, which has little effect on the final performance of the device with simulation analysis.

**Figure 8:**
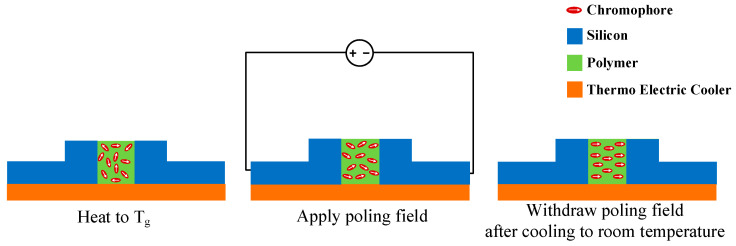
Schematic diagram of poling EO polymer on chip.

As, initially, the orientation of chromophores in the Slot waveguide follows thermodynamic statistical rules, it implies that there is no macroscopic EO response. To achieve a macroscopic EO effect in the material, it is necessary to align the chromophores in the EO polymer material. The most common method used for this purpose is electric field poling [30], which is divided into corona poling and contact poling. The on-chip poling of EO polymers is implemented directly by using the electrodes of the modulator and its electric field poling mechanism is similar to that of contact poling. As shown in Figure 8, the electric field poling mechanism of EO polymers can be described as follows: at the glass transition temperature (T_g_), the polymer transitions from a glassy state to a high-elasticity state and, at this point, the binding force between the chromophores and the polymer is relatively weak, allowing the chromophores to rotate freely within a certain range in the polymer. Therefore, under the external electric field, the dipole moment of the chromophore tends to align with the electric field direction, causing the chromophores to rotate and maintain their alignment for a period of time. When this rotation becomes stable, the temperature of the polymer is cooled to room temperature and the polymer returns to a state of strong binding force. The chromophores in the polymer are thus unable to rotate freely after the external electric field is removed, resulting in their maintained alignment. Through the above series of operations, the polarized polymer achieves a macroscopic EO effect.

For on-chip poling of EO polymers, a DC probe is connected to the modulator electrode PAD after the chip is fixed by a vacuum adsorption plate and a DC high-voltage power supply (Teslaman, TD2200P5-150, Dalian, Liaoning, China) is used to provide an adjustable DC voltage to the chip. A source meter (Keithley, 2602A, Beaverton, OR, USA) is used to monitor the current through the polymer in the Slot during poling. The temperature on chip is controlled by a temperature controller (Lightwave, LDT-5525B, Irvine, CA, USA) during poling and the chip can be rapidly heated and cooled by a semiconductor cooler.

**Figure 9:**
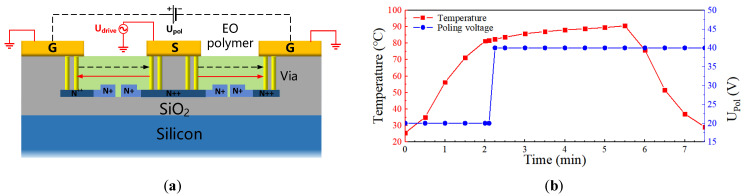
(**a**) Cross-section schematic diagram of the modulation area with poling DC voltage; (**b**) voltage (blue) and temperature (red) during on-chip poling of EO organic material.

Figure 9a shows a schematic diagram of the electric field in the cross-section of the modulating region during device polarization and operation. The electrodes used for polarization are the same as those used for modulation, which simplify device structure and reduce preparation difficulty. The black (dotted line) and red (solid line) arrows in the figure, respectively, indicate the direction of the polarization electric field and the modulation electric field in the cross-section of the modulating region, and the directions of the modulation electric field and polarization electric field in the two arms are the same and opposite, respectively. This makes a push–pull operation possible and improves the modulation efficiency and reduces power consumption of the device [31]. In the polarization process, a large polarization voltage is required so that the polarized EO polymer has a high EO coefficient, resulting in a small half-wave voltage length product. However, higher polarization voltage can cause dielectric breakdown and scorch the EO polymer. Figure 9b shows the optimized voltage and temperature curves on the chip for the polarization process. During the polarization process, the pre-polarization voltage is set to 20 V until the temperature reaches 82 °C (glass transition temperature T_g_). After the temperature reaches T_g_, the polarization voltage gradually increases to 40 V. About three minutes later, while maintaining the voltage at 40 V, the temperature on the chip rapidly drops to room temperature and, finally, the polarization voltage is withdrawn to complete the polarization. The entire polarization process takes about seven minutes.

## 3. Device Characterization

The S-parameters of electrode directly affect the final bandwidth and power consumption of EO modulator. When measuring its electrical performance, such as S-parameters, it can be considered as an electrical two-port network and analyzed directly by an electrical vector network analyzer (VNA, Keysight, N5247B, Santa Rosa, CA, USA). In order to accurately measure the performance parameters of the modulator, the reference plane of the VNA should be calibrated to both ends of the electrode before the measurement. The calibration method used is the Short-Open-Load-Through (SOLT) method, which is one of the most dominant calibration methods for on-chip testing [32].

**Figure 10:**
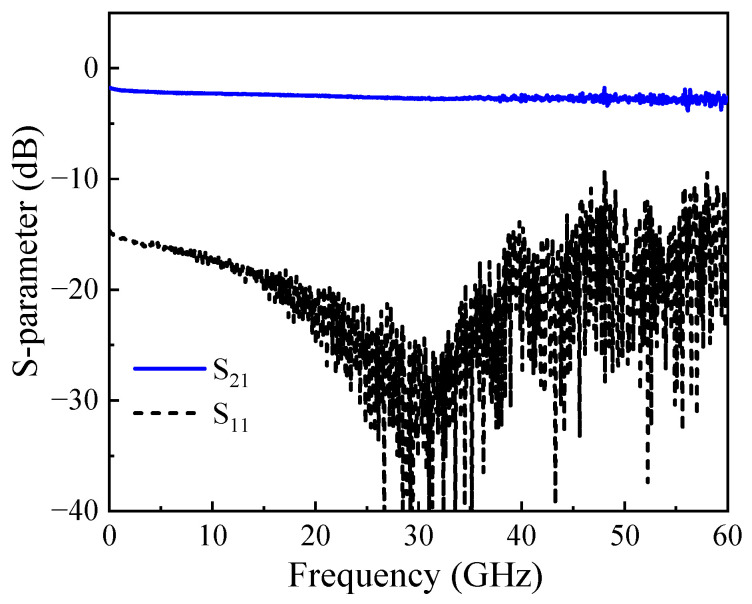
S-parameters of modulator travelling-wave electrode.

The measured S-parameters of the electrode are shown in Figure 10. The S_21_ curve decreases by approximately 1 dB over the range of 60 GHz, while the modulator’s S_11_ is less than −10 dB, indicating low transmission loss of the modulator electrodes and small reflected signals. According to Equations (1) and (2), the characteristic impedance of electrode Z_0_ and propagation constant γ can be calculated by S-parameters [33].

(1) $$Z_{0}=Z_{ref}\sqrt{\frac{{\left(1+S_{1}{}_{1}\right)}^{2}-S_{21}^{2}}{{\left(1-S_{1}{}_{1}\right)}^{2}-S_{21}^{2}}}$$

(2) $$e^{-}{}^{\gamma l}=\{\frac{1-S_{11}^{2}+S_{21}^{2}}{2S_{21}}\pm {\left[\frac{{\left(S_{11}^{2}-S_{21}^{2}+1\right)}^{2}-{\left(2S_{11}\right)}^{2}}{{\left(2S_{21}\right)}^{2}}\right]}^{\frac{1}{2}}\}$$

(3) $$\gamma =\alpha +i\frac{\omega N_{\mathrm{RF}}}{c}$$

where *Z_ref_* is the reference impedance of the measurement system, which is 50 ohms. *α* is the attenuation factor, *N*_RF_ is the effective refractive index of the microwave signal, and *c* is the speed of light in a vacuum.

**Figure 11:**
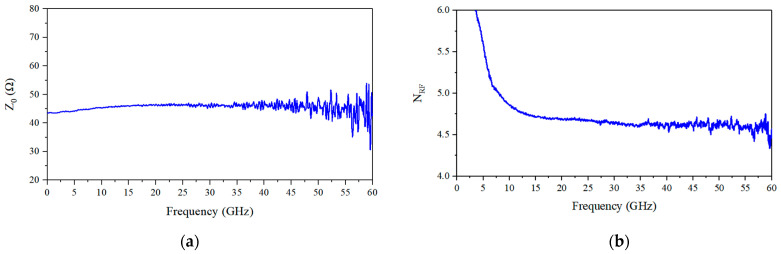
Distributed parameters of electrode obtained by S-parameters: (**a**) electrode characteristic impedance; (**b**) microwave effective refractive index.

The characteristic impedance and refractive index curves of the coplanar waveguide electrode are shown in Figure 11a and Figure 11b, respectively. The characteristic impedance of the electrode is around 45 ohms, while the effective refractive index of the microwave is around 4.7.

**Figure 12:**
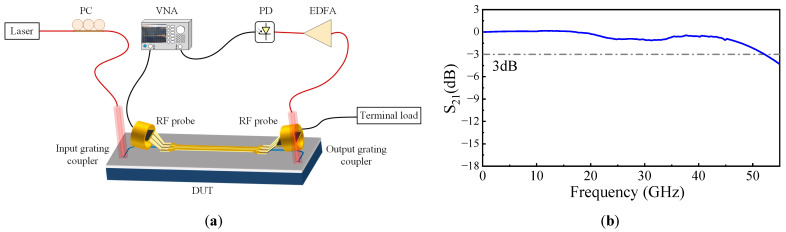
(**a**) Experimental setup for measuring RF S21 response; (**b**)measured EO S_21_ of the modulator with modulation length of a 0.5 mm.

For high-performance EO modulators, the frequency response bandwidth is the most important factor determining signal transmission speed. The experimental setup is shown in Figure 12a. The RF signal from VNA port 1 is loaded into the modulator chip under test through the RF probe, and the optical signal output by the modulator is amplified by an erbium-doped fiber amplifier (EDFA, Keopsys, CEFA-C-HG-SM-40-B130, Lannion, France) and input into the photodetector (PD, Finisar, XPDV3120R, San Jose, CA, USA), and the electrical signal output by the PD is returned to VNA port 2 to obtain the S_21_ curve of the chip to be tested. An RF probe is used to connect a 50 Ω terminal load to the modulator chip. The test results are shown in Figure 12b. It can be seen that the EO modulator with a 3 dB bandwidth of over 50 GHz, where the frequency response of the RF probes and PD has been removed.

The half-wave voltage length product *V*_π_*L* characterizes the modulating efficient of EOM for a specific wavelength of input light. The smaller *V*_π_*L* means that the lower power consumption and more compact device size. It is dependent on:

(4) $$V_{\pi }L=\frac{\lambda W_{Slot}}{2n^{3}\gamma_{33}\Gamma }$$

where the wavelength *λ* of laser is 1550 nm, the refractive index of the polymer material *n* is 1.72, the width of Slot waveguide *W_Slot_* is 150 nm, and the *γ*_33_ is the in-device EO coefficient. Γ is the confinement factor of the optical field, defined as the ratio between the optical power in the Slot waveguides and the total power propagating, as shown in Equation (5). The confinement factor Γ for the tested MZM was calculated to be approximately 14.2%.

(5) $$\Gamma =\frac{\iint_{Slot}{\left|E\left(x,y\right)\right|}^{2}dxdy}{\iint {\left|E\left(x,y\right)\right|}^{2}dxdy}$$

**Figure 13:**
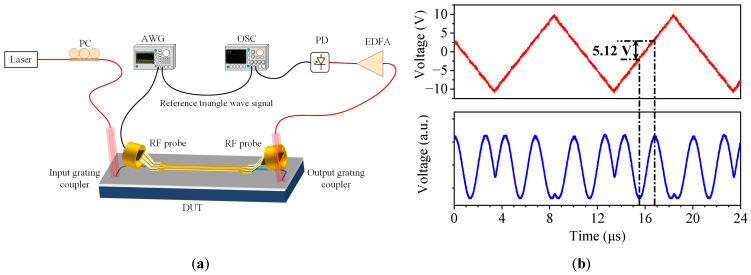
(**a**) Experimental setup for measuring half-wave voltage; (**b**) measured half-wave voltage of the modulator with modulation length of 0.5 mm.

The half-wave voltage *V*_π_ of the modulator is obtained by measuring the EO response when the driving voltage of the triangle wave signal increases linearly with time. The experimental setup is shown in Figure 13a. In the experiment, an arbitrary waveform generator (RIGOL, DG4202, Suzhou, Jiangsu, China) is used to provide a triangular wave electrical signal loaded onto the modulator chip under test, and the output optical signal is converted into an electrical signal by PD, and the input and output signal waveforms are compared in real time by using a dual-channel oscilloscope (RIGOL, DS1104, Suzhou, Jiangsu, China) to obtain the half-wave voltage *V_π_* of the modulator chip. Figure 13b shows that the measured half-wave voltage *V_π_* at 100 kHz is 5.12 V and the half-wave voltage length product *V*_π_*L* is 0.26 V·cm, with the modulation length of 0.5 mm. According to (4), the in-device EO coefficient *γ*_33_ is calculated to be 63 pm/V, which is about twice that of LN.

**Figure 14:**
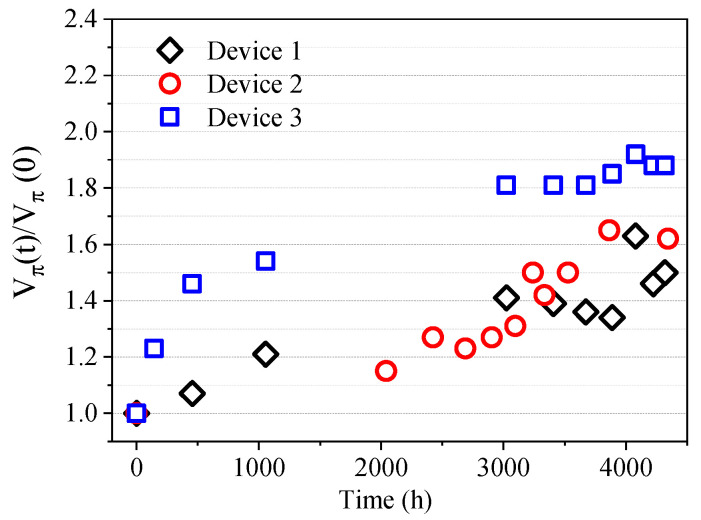
Time stability of devices at room temperature.

To test the stability of the device modulation performance, three sample chips were tracked over a long period of time, as shown in Figure 14. The half-wave voltage of the device rises to 1.4–1.8 times its initial value after more than 4000 h, and the thermo-optical coefficient of the polymer material decreases to about 0.6–0.7 just after poling and remains stable. This indicates that the device still maintains a comparable modulation performance after being stored at room temperature for more than 4000 h to meet the practical use requirements.

## 4. Microwave Phase Shifter Based on Dual-Parallel Mach–Zehnder Modulator

**Figure 15:**
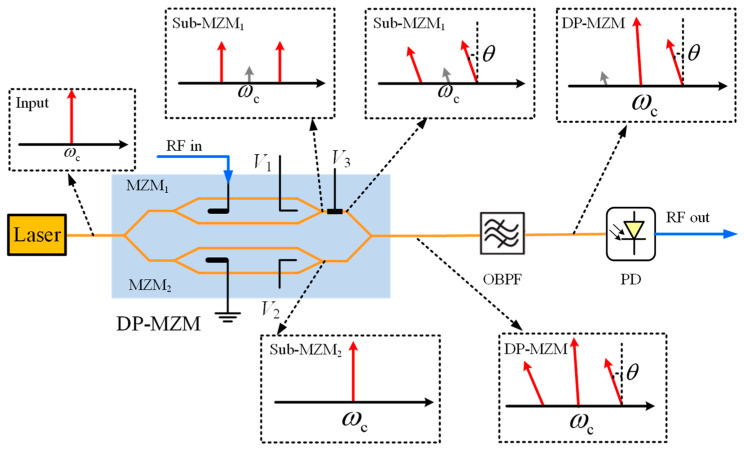
Schematic diagram of microwave phase shifter based on DP-MZM.

Due to the numerous benefits, including wide bandwidth, low losses, and immunity to electromagnetic interferences, photonics RF phase shifter has been an important component of phased-arrayed beam-forming network and microwave photonic filters. We demonstrate a simple photonics RF phase shifter based on SOH Dual-Parallel Mach–Zehnder modulator (DP-MZM). The schematic diagram is shown in Figure 15.

DP-MZM is comprised of two MZMs that can input RF signal individually in parallel. And the optical phase between two sub-MZMs can be controlled by *V*_3_. The laser output optical carrier enters the DP-MZM and is divided into two equal power parts into two sub-MZMs (sub-MZM_1_ and sub-MZM_2_). Sub-MZM_1_ is loaded with RF signal and operates at the minimum point to achieve carrier suppression double-sideband modulation. The carrier power in the output spectrum of Sub-MZM_1_ reaches minimum and the first-order sideband power reaches maximum, and the power magnitude of the carrier is determined by the extinction ratio of the modulator. The Sub-MZM_2_ is not loaded with RF signal and only provides optical carrier, working at the maximum point. Finally, the relative phase between the two sidebands from Sub-MZM_1_ and carrier from Sub-MZM_2_ is adjusted by *V*_DC3_ and then combined to output. Then, DP-MZM output signal is filtered by optical filters to remove the negative first-order optical sidebands from the spectral components and input to the photodetector. The photodetector transforms the signal into electric domain. Neglecting the higher-order components of the spectrum, the DP-MZM output signal can be expressed as follows when only the first-order optical sidebands are considered:

(6) $$E_{1}=k_{1}A_{0}e^{i(\omega_{c}t+\theta )}+A_{0}e^{i\omega_{c}t}+A_{1}e^{i\left[(\omega_{c}\pm \omega_{RF})t+\theta \right]}$$

where *A*_0_ is the amplitude of the sub-MZM_2_ output optical carrier. *k*_1_^−2^ is the carrier suppression ratio, which represents the ratio of the residual carrier power when sub-MZM_1_ is operating at the minimum point and the maximum output optical power. It should be noted that the value of *k*_1_^−2^ is determined by the modulator extinction ratio. *A*_1_ is the amplitude of the positive and negative first-order sidebands when sub-MZM_1_ is operating at the minimum point. *θ* is the phase difference caused by the DC bias voltage *V*_3_ and the *θ* = π·(*V*_3_/*V*π_3_)^2^. *V*π_3_ is the DC voltage of π phase shift.

Before entering the PD, the negative first-order sidebands need to be filtered out by using an optical bandpass filter. The power of the optical carrier is also reduced a little due to the restricted steepness of the optical bandpass filter, and the spectral components after the optical filter can be expressed as:

(7) $$E_{2}=k_{2}A_{0}e^{i\omega_{c}t}+k_{2}k_{1}A_{0}e^{i(\omega_{c}t+\theta )}+A_{1}e^{i\left[(\omega_{c}+\omega_{RF})t+\theta \right]}+k_{3}A_{1}e^{i\left[(\omega_{c}-\omega_{RF})t+\theta \right]}$$

where *k*_2_^−2^ represents the power attenuation of the optical carrier after the filter; *k*_3_^−2^ is the power ratio of the negative first-order optical sidebands to the positive first-order optical sidebands. After the PD photoelectric conversion of the photocurrent from these spectral components beat frequency, ignoring the DC term as well as the multiplier term, the photocurrent can be expressed as:

(8) $$\begin{array}{c} i\propto E_{2}\cdot E_{2}^{*}\\ \;\propto k_{2}A_{0}A_{1}\cos(\omega_{RF}t+\theta )+k_{2}k_{3}A_{0}A_{1}\cos(\omega_{RF}t-\theta )\\ \;+k_{2}k_{1}A_{0}A_{1}\cos(\omega_{RF}t)+k_{3}k_{2}k_{1}A_{0}A_{1}\cos(\omega_{RF}t) \end{array}$$

We would like to have only cos(*ω_RF_t + θ*) in the PD output signal. If the negative first-order sidebands are completely filtered out and the sub-MZM_1_ does not output an optical carrier at all, the phase difference between the output microwave signal and the input microwave signal depends entirely on the phase difference *θ* between the two sub-MZMs. But there are other terms in the output signal due to the finite extinction ratio of the sub-MZM_1_. Also, the negative first-order sidebands cannot be completely filtered out, especially when the sidebands are closer to the carrier, which results in the presence of the correlation term in the output signal. Equation (8) is further simplified to:

(9) $$i\propto A_{3}\cos(\omega_{RF}t+\phi_{1})+k_{3}A_{3}\cos(\omega_{RF}t-\phi_{1})$$

where *A_3_* and *ϕ*_1_ are:

(10) $$A_{3}=A_{0}A_{1}k_{2}\sqrt{1+k_{1}^{2}+2k_{1}cos\theta }$$

(11) $$\phi_{1}=\arctan(\frac{\sin\theta }{\cos\theta +k_{1}})$$

The output microwave signal can eventually be expressed as:

(12) $$i\propto A\cos(\omega_{RF}t+\phi )$$

where *A* and *ϕ* are:

(13) $$A=A_{3}\sqrt{1+k_{3}^{2}+2k_{3}cos2\phi_{1}}$$

(14) $$\phi =\arctan(\frac{\left(1-k_{3}\right)\sin\phi_{1}}{\left(1+k_{3}\right)\cos\phi_{1}})$$

**Figure 16:**
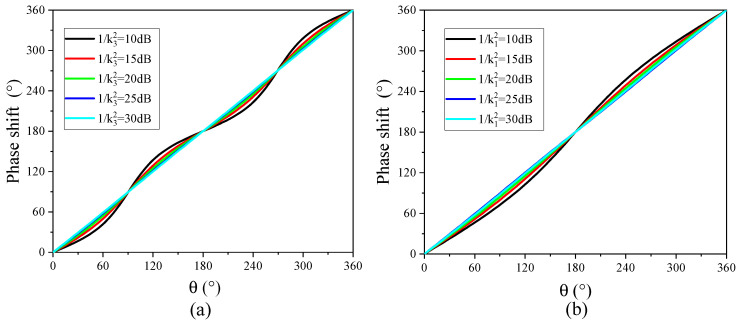
The variation in the RF phase shift with *θ*: (**a**) 1/$k_{1}^{2}$ = 40 dB, $k_{3}^{2}$ = [10 dB, 15 dB, 20 dB, 25 dB, 30 dB]; (**b**) 1/$k_{1}^{2}$ = [10 dB, 15 dB, 20 dB, 25 dB, 30 dB], 1/$k_{3}^{2}$ = 40 dB.

**Figure 17:**
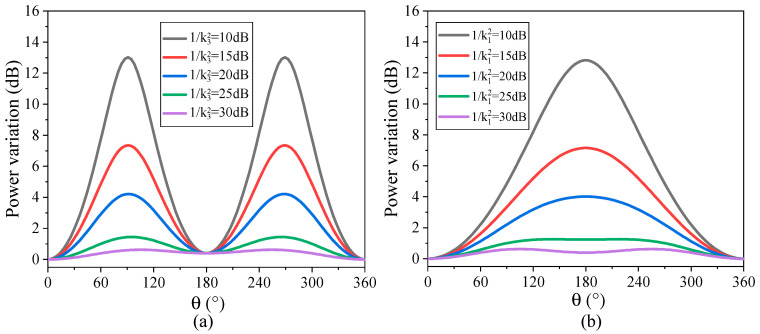
The variation in the RF power with θ: (**a**) 1/$k_{1}^{2}$ = 40 dB, $k_{3}^{2}$ = [10 dB, 15 dB, 20 dB, 25 dB, 30 dB]; (**b**) 1/$k_{1}^{2}$ = [10 dB, 15 dB, 20 dB, 25 dB, 30 dB], 1/$k_{3}^{2}$ = 40 dB.

The variations in the RF phase shift and the power with *θ* for different carrier suppression ratios *k*_1_^−2^ and sideband suppression ratios *k*_3_^−2^ are calculated by Equation (12), as shown in Figure 16 and Figure 17. As can be seen from Figure 16, the phase shift of the RF signal basically presents a linear increase trend with the increase in phase difference *θ*, and the linearity of phase shift increase is better with the increase in carrier suppression ratio and sideband suppression ratio. Figure 17 shows the variation in RF signal power with phase difference. As can be seen from Figure 17, in the range of phase difference changes, the power variation in the RF signal presents periodic fluctuations. When the carrier suppression ratio is 40 dB (30 dB) and the sideband suppression ratio is 30 dB (40 dB), the RF signal power variation fluctuation is only in the range of 0.6 dB.

In the experiments, the laser source (NKT Photonics K81-152-04, Birkerød, Denmark) outputs an optical carrier with optical power of 9 dBm. The optical signal is adjusted by polarization controller and then couples through the waveguide grating into the modulator chip. VNA port 1 outputs microwave signal to sub-MZM1, which operates at the minimum bias point to obtain the carrier-suppressed double-sideband modulated optical signal. The phase difference between sub-MZM_1_ and sub-MZM_2_ is adjusted by a thermo-optic electrode. The optical signal, through the waveguide grating coupling, moves out of the chip into the EDFA. The amplified signal is input to the optical bandpass filter (OPBF, Finisar, WaveShaper 16000S, San Jose, CA, USA) to suppress the negative first-order sidebands and then input to the photodetector. The photodetector transforms the optical signal to an electric signal and returns it to port 2 of the VNA. According to the S-parameters, the VNA obtains the amplitude and phase shift of the RF signal.

**Figure 18:**
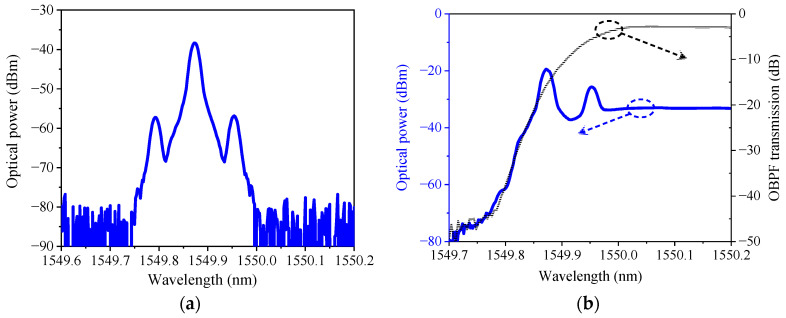
(**a**) Output optical spectrum of DP-MZM; (**b**) the modulated optical signal after OBPF (blue curve) and the OBPF transmission response (black curve).

In experiment, the sub-MZM_1_ operates at the minimum point and sub-MZM_2_ operates at the maximum point. The optical bandpass filter is set to make a lower sideband at the stopping band and the carrier and upper sideband at the passing band. The output optical spectrum of DP-MZM measured by optical spectrum analyzer (YOKOGAWA, AQ6370D, Tokyo, Japan) is shown in Figure 18a. The modulated optical signal after OBPF and the transmission spectrum of OBPF are shown in Figure 18b.

**Figure 19:**
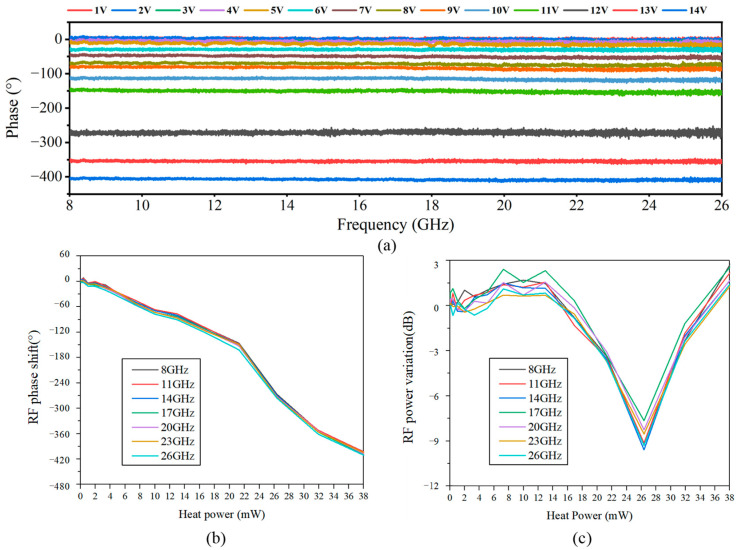
Measurement results of photonic RF phase shifter: (**a**) measured phase shifts for different heating powers over a frequency range from 8 GHz to 26 GHz; (**b**) variation in RF phase shift with heat power at different RF frequencies; (**c**) variation in RF power with heat power at different RF frequencies.

Figure 19a shows the tunable RF phase up to 410° over the frequency from 8 to 26 GHz. And the RF phase shift and power stability versus the heating power for different RF frequencies of 8–26 GHz are measured and shown in Figure 19b,c. The photonic RF phase shifter based on DP-MZM achieves a phase shift range of greater than 410° at a phase shift power of 38 mW and has a well-in-band consistency. But the RF power has a variation during phase shift. From Figure 16 and Figure 17, it can be seen that the power variation is mainly due to the finite carrier suppression ratio. According to the theoretical analysis in Figure 17, the carrier suppression ratio of sub-MZM_1_ can be presumed to be higher than 10 dB.

## 5. Conclusions

We designed and fabricated an SOH Mach–Zehnder modulator featuring an EO response 3 dB bandwidth of 50 GHz and half-wave voltage length product of 0.26 V·cm, by virtue of the high EO coefficient in the device of *γ*_33_ = 63 pm/V. In addition, we investigated a photonic RF phase shifter based on the SOH dual parallel Mach–Zehnder modulator. A phase shift range of 410° was achieved over the frequency band from 8 GHz to 26 GHz with a power consumption of less than 38 mW.

## Data Availability

Data underlying the results presented in this paper are not publicly available at this time but may be obtained from the authors upon reasonable request.
